# Temperature at parental generation affects bacterial communities associated with offspring for both host and parasitoid

**DOI:** 10.1093/femsec/fiag046

**Published:** 2026-05-01

**Authors:** Camila Souza Beraldo, Diego Castillo Franco, Saskya van Nouhuys, Anne Duplouy

**Affiliations:** Organismal and Evolutionary Biology Research Programme, Faculty of Biological and Environmental Sciences, University of Helsinki, Helsinki 00790, Finland; Department of Molecular Biosciences, University of Kansas, Lawrence, KS 66045, United States; Department of Developmental Biology and Morphology of Invertebrates, Institute of Zoology and Biomedical Research, Faculty of Biology, Jagiellonian University, Kraków 30-387, Poland; Department of Biology, Faculty of Sciences, Ghent University, Ghent 9000, Belgium; Centre for Ecological Sciences, Indian Institute of Science, Bengaluru560012, India; Organismal and Evolutionary Biology Research Programme, Faculty of Biological and Environmental Sciences, University of Helsinki, Helsinki 00790, Finland; Institute for Life Science (HiLIFE), Faculty of Biological and Environmental Sciences, University of Helsinki, Helsinki 00790, Finland

**Keywords:** trophic interactions, koinobiont, microbiota, microbial symbiosis, *Wolbachia*, climate change

## Abstract

The thermal conditions experienced during development can affect host-associated microbial communities. We still know little about whether such effects similarly persist across life stages between different species. In particular, it is unclear if the bacterial communities of closely interacting species, such as hosts and their endoparasitoids, exhibit similar responses to thermal conditions. We reared two generations of the *Melitaea cinxia* butterfly and its specialised parasitoid wasp, *Hyposoter horticola*, at three temperatures in the laboratory (26°C, 28°C, and 31°C). We found that the two species harbour different bacterial communities as adults, with the parasitoid exhibiting higher bacterial richness than its host butterfly. When the parental generation of the butterfly was exposed to high temperatures, the F1 generation exhibited increased bacterial richness but a reduced diversity (Shannon index). The opposite effect was observed for its parasitoid, but only for the wasps infected with *Wolbachia*, which appears sensitive to thermal conditions. Collectively, these results highlight that the bacterial communities of insect hosts and their parasitoids are distinct units, differently susceptible to environmental thermal conditions, particularly to temperatures experienced at the parental generation.

## Teaser

Insect hosts and their parasitoids harbour distinct microbiota that respond differently to thermal conditions, with parental temperatures and *Wolbachia* infection partly shaping these effects.

## Introduction

The microbial communities associated with insects, commonly referred to as the microbiota, encompass communities of bacteria, archaea, fungi, protozoa, and microalgae (Berg et al. 2020), which are structured by biotic and abiotic factors (Douglas 2015). The host identity often plays a key role (Malacrinò 2022), with the microbiota often reflecting the ecological niche and the dietary specialisation of each insect species (Ludington 2024). For example, symbiotic microbes can supplement their aphid and bedbug hosts’ diet with essential nutrients, such as amino acids and vitamins (Hansen and Moran 2011, Serrato-Salas and Gendrin 2023), or contribute to digestion and detoxification by herbivorous caterpillars and beetle larvae (Jing et al. 2020). Diet composition itself can also strongly shape microbial communities; for example, in honeybees, a pollen-based diet has been shown to support more diverse bacterial communities than a nectar-based diet (Kešnerová et al. 2020). Additionally, shifts in the microbiota of insects occur throughout their life cycle and development. Although early life microbial colonisation can determine the associated microbial community composition in later stages (Sprockett et al. 2018, Debray et al. 2022), each life stage presents distinct physiological and nutritional demands, which can drive changes in microbial communities (English and Barreaux 2020, Kingsolver and Buckley 2020, Brueggemann et al. 2025). This is particularly evident in holometabolous insects, which undergo complete metamorphosis through the larval, pupal, and adult stages. For example, in the monarch butterfly (*Danaus plexippus*), bacterial communities are dominated by *Pantoea* in caterpillars, *Enterobacter* in pupae, and *Asaia* in adults (Sanaei et al. 2024).

Temperature is a key abiotic factor affecting insect-associated microbial communities (Moghadam et al. 2018, Sepulveda and Moeller 2020, Iltis et al. 2022). In the laboratory, *Drosophila melanogaster* flies reared at cool temperatures (13°C) exhibit more diverse bacterial communities than those reared under warmer conditions (31°C). Similarly, high temperatures have been shown to negatively affect the titer of the bacterial symbiont *Wolbachia* in *Drosophila* spp. (Lau et al. 2020, Hague et al. 2022); and differences in *Wolbachia* titer are known to have cascading effects on the abundance of other bacterial families, including Spiroplasmataceae, Lactobacillaceae, and Acetobacteraceae (Simhadri et al. 2017, Fromont et al. 2019). Such shifts in the host-associated bacterial composition can lead to functional changes, with potential consequences for host reproduction and survival (Werren et al. 2008), and for the fitness of their offspring (Funkhouser and Bordenstein 2013). Although the transgenerational effects of parental environmental conditions are common in various insect life-history traits (Saastamoinen et al. 2013, Sánchez-Tójar et al. 2020), how temperature experienced at any life stage may impact the composition and species richness of bacterial communities associated with subsequent life stages or generations of the insect hosts remains generally unclear.

Koinobiont endoparasitoids are insects that lay their eggs inside other arthropods, with larvae developing within the host while allowing the host to continue growing until parasitoid pupation, which ultimately results in host death (Godfray 1994). Although adult parasitoids are free-living, this intimate developmental association creates prolonged physiological and ecological interaction between host and parasitoid. Studies on endoparasitoid-associated bacterial microbiota suggest that hosts and their endoparasitoids can harbour similar communities. For example, parasitoid larvae of the species *Cotesia vestalis* and *Diadromus collaris* have been shown to share similar gut bacterial communities with each other and with their shared lepidopteran host, *Plutella xylostella* (Hu et al. 2024). Such similarity may arise because endoparasitoids develop within host tissues, and are exposed to host-associated microbial communities and metabolites. This may facilitate microbial transfer or convergence toward shared microbial pools if microbes persist across metamorphosis (Gao et al. 2021, Gloder et al. 2021, Hu et al. 2024). Conversely, divergence in parasitoid–host microbiota may result from species-specific physiological and immune filtering, extensive microbial loss during complete metamorphosis, or differences in adult diet and behaviour (Hammer et al. 2014, Malacrinò 2022, Manthey et al. 2023). Although several studies have compared the bacterial microbiota of parasitoids and their hosts (Gao et al. 2021, Gloder et al. 2021, Ashraf et al. 2022, Wang et al. 2023, 2024, Gwokyalya et al. 2024, Hu et al. 2024), the stability of these host–parasitoid microbiota relationships across environmental contexts and life stages remains unclear. In particular, environmental stressors, including temperature experienced during development or across generations, may alter bacterial assembly processes, potentially reinforcing species-specific filtering or, alternatively, promoting parallel responses in hosts and parasitoids through shared microbial pools or constrained physiological responses. Direct comparisons of host and parasitoid bacterial microbiota under controlled and ecologically relevant environmental conditions are therefore needed to demonstrate how environmental history interacts with intimate biotic interactions to structure insect-associated bacterial communities.

The Glanville fritillary butterfly, *Melitaea cinxia* (Linnaeus, 1758) (Lepidoptera: Nymphalidae), its specialist parasitoid wasp *Hyposoter horticola* (Gravenhorst, 1829) (Ichneumonidae: Campoplaginae) and their primary host plant, *Plantago lanceolata* (Plantaginaceae), are found across Europe and northern Asia (Kuussaari et al. 2004, van Nouhuys and Hanski 2004, Couchoux et al. 2016). In the Åland Islands, Finland, these species and their interactions have been the focus of extensive ecological and evolutionary research over the past three decades (Ehrlich and Hanski 2004, Duplouy et al. 2015, Opedal et al. 2020). Both the host butterfly and the endoparasitoid wasp are univoltine in northern Europe. Butterfly eggs are laid in clutches in early summer, and the caterpillars develop in gregarious sibling groups throughout summer and fall. They diapause in groups over the winter and then continue to feed until pupation in the spring (Kuussaari et al. 2004). *Hyposoter horticola* parasitise newly formed *M. cinxia* caterpillars just before they hatch from the eggs. The parasitoid larvae then develop within caterpillar hosts until just before pupation occurs in the following spring (van Nouhuys and Lei 2004).

Although the temperate maritime climate of the Åland Islands is characterised by cool summers and mild winters, in recent years, the region has experienced increasing temperatures and more frequent droughts (Kahilainen et al. 2022). High temperatures and drought have been associated with a reduction in survival and fitness in *M. cinxia* caterpillars due to desiccation and changes in host plant quality (Nieminen et al. 2004, Tack et al. 2015, Salgado et al. 2020). The environmental change affects the dynamics of the parasitoid population primarily through its effects on the dynamics of the host butterfly population dynamics (Nair et al. 2018).

The bacterial community of *M. cinxia* has been suggested to be transient and facultative, acquired primarily from the environment, rather than consisting of resident and obligate endosymbionts (Minard et al. 2019). The composition of the bacterial microbiota of *M. cinxia* caterpillars has been shown to differ depending on the host food plant species, environmental conditions, and exposure to pathogens (Minard et al. 2019). For example, fungal or viral infections in host plants across Åland can alter the microbial communities available to *M. cinxia* caterpillars, potentially influencing their health and development (Laine 2004, Susi and Laine 2021). The bacterial microbiota of *H. horticola* remains largely uncharacterized other than the vertically transmitted endosymbiotic bacterium *Wolbachia* (Duplouy et al. 2015, 2021; van Nouhuys et al. 2016). In Åland, this infection persists at a stable intermediate level of about 50% of the population across the years (Duplouy et al. 2015, 2021), likely due to a balance of costs and benefits of infection (Karisto et al. 2022). The bacterium is not found in the host butterfly (Jones et al. 2025). Minard et al. (2019) showed that *M. cinxia* caterpillars containing *H. horticola* larvae had a different bacterial microbiota than unparasitized caterpillars, likely reflecting a mixture of bacterial communities from both the caterpillar and the *Wolbachia*-infected developing *H. horticola* larvae.

Here, we investigated how parasitoidism and temperature conditions across generations and development influence the insect-associated bacterial community. We exposed two generations of the butterfly *M. cinxia* and its specialist parasitoid *H. horticola* to three rearing temperatures in a cross-sectional experimental setup. We addressed two questions: (i) How does the bacterial community differ between a butterfly host and its specialist parasitoid at the adult stage? and (ii) How do temperature conditions experienced by the parents and across developmental stages (prediapause vs. postdiapause caterpillar stages) influence the bacterial communities of adult offspring of these two interacting species? Because *H. horticola* is a specialist parasitoid of *M. cinxia* caterpillars, the intimate developmental association of the parasitoid larvae with the host caterpillar may promote similarity in bacterial microbiota between host and parasitoid through shared microbial exposure. Under this scenario, thermal stress would be expected to induce similar changes in the bacterial microbiota of F1 individuals of both species. Alternatively, host and parasitoid bacterial microbiota may diverge as a result of species-specific physiological, immunological, or ecological filtering, such that thermal stress affects the bacterial community of each species independently. Through these contrasting expectations, we assess whether shared ecology or species identity plays the dominant role in structuring the bacterial microbiota in this host–parasitoid system.

## Material and methods

### Experimental design

In the laboratory, *M. cinxia* was fed exclusively on *P. lanceolata* plants grown at 25°C in the greenhouse. The plants were fertilised with 100 ml of 0.02% Neko NPK solution once a week. The plants were also watered and sprayed with soap and sodium bicarbonate solution 3 days a week to avoid aphids and fungal infestations. All pre- and postdiapause caterpillars were provided *ad libitum P. lanceolata* leaves on a daily basis, while adult insects were fed 30% honey water on a sterilised sponge once a day.

#### Parental generation *(F0)*

**Figure 1:**
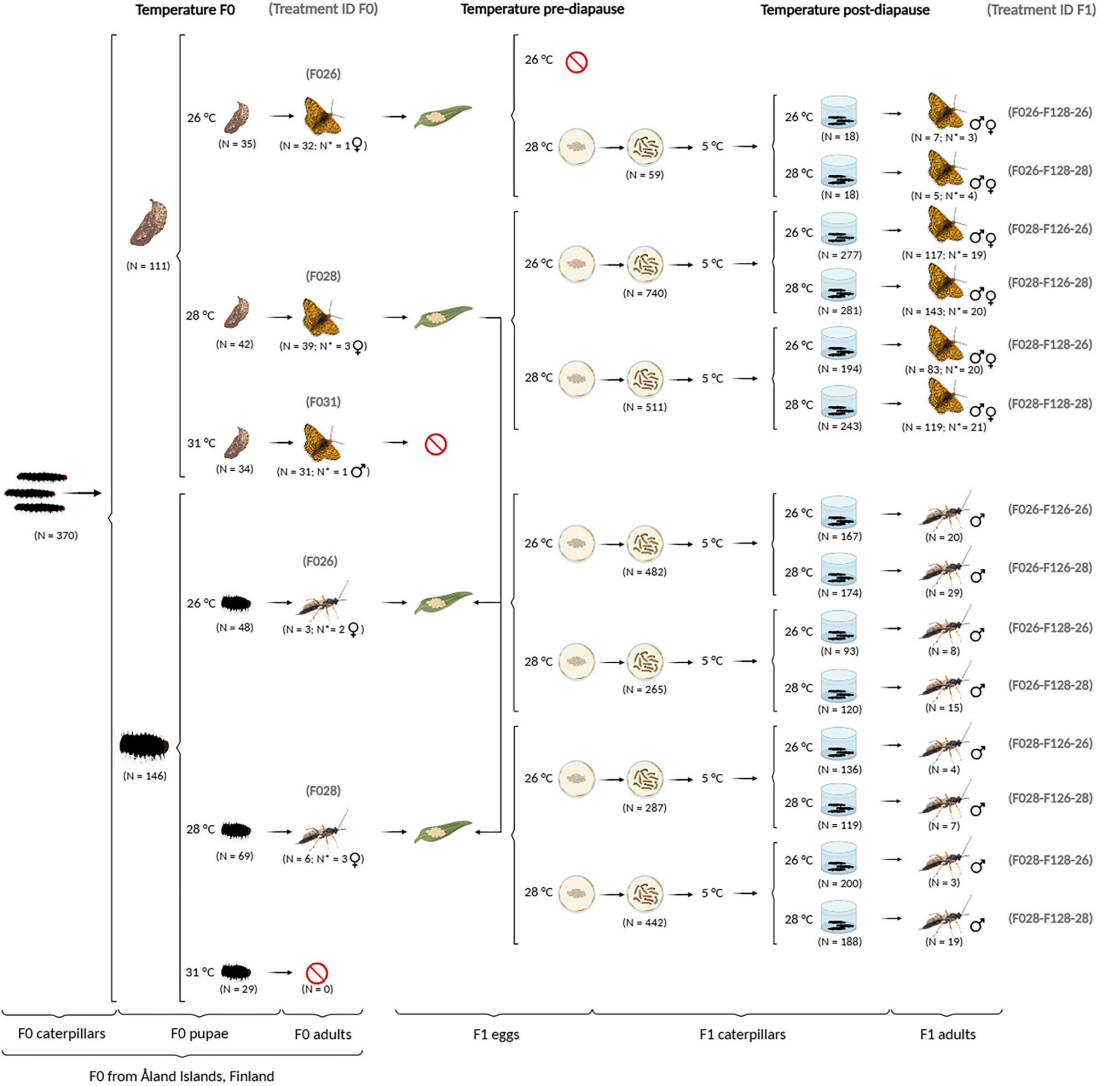
Cross-sectional experimental design used over the parental (F0) and offspring (F1) generations. The F0 caterpillars were collected in the Åland Islands, Finland, in the fall of 2020, and the F1 generation was born and reared under laboratory conditions. N represents the total number of individuals that survived in each developmental stage, while N* represents the number of individuals selected for DNA extraction. All adult *H. horticola* specimens were used for DNA extraction (N = N*).

The first generation (F0) of *M. cinxia* (*N* = 370) was collected in the Åland archipelago, southwest Finland, in the fall of 2020, when the caterpillars were in the early stage of diapause. All specimens received a unique identifier upon collection in the field (Ojanen et al. 2013). The diapausing *M. cinxia* caterpillars were kept at 5°C, 24 h dark, with constant 80% humidity in an incubator (Climacell 222 L, MMM Group), from early 2020 fall until the following spring 2021. To break the diapause, caterpillars were exposed to a 12:12 h light:dark cycle, followed by a gradual increase in temperature over 3 days: from 15°C:8°C day:night temperatures, to 20°C:13°C, and finally 28°C:18°C. The prediapause caterpillars were then individually reared in plastic containers at 28°C:18°C. About one-third of these caterpillars were naturally parasitised by *H. horticola*, some of which were further hyperparasitized by *Mesochorus* cf. *stigmaticus* (van Nouhuys and Hanski 2004, Montovan et al. 2015). When caterpillars or parasitoid larvae pupated, they were split into three temperature treatments until the emergence of adults: 26°C:18°C (mean = 22°C; F026), 28°C:18°C (mean = 23°C; F028), or 31°C:18°C (mean = 24.5°C; F031) (Fig. 1). All experimental temperatures are representative of a range of microclimatic conditions that caterpillars experience naturally at the ground level in Åland during spring and summer (Kuussaari et al. 2004, Saastamoinen 2007, Duplouy and Hanski 2015). Upon emergence, adult butterflies were labelled on their wings with a unique number and moved to 27 L mesh cloth cages (with a maximum of 10 individuals per cage, separated by sex), while adult wasps were kept in individual 100 ml containers, inside incubators and under their respective temperature treatment. Adult female *H. horticola* wasps reach reproductive maturation after 10–12 days (Montovan et al. 2015, Couchoux et al. 2016).

*Melitaea cinxia* butterflies mated under natural sunlight in the greenhouse (average temperature was 25°C). We recorded the IDs of each mating pair, and then the mated females were each offered a potted host plant to lay eggs on. Plants carrying egg clutches were set aside and kept at 25°C for a week. Then, when caterpillars were nearly ready to hatch from the eggs, they were offered for parasitisation by manually placing a mature F0 female *H. horticola* wasp (*N* = 9) on the *M. cinxia* egg clutch using a mouth aspirator (Montovan et al. 2015). We observed oviposition by each wasp to ensure parasitism occurred. *Hyposoter horticola* has been described to not mate in the lab, therefore, all female wasps remained unmated and thus laid only unfertilized haploid eggs, which developed into males through parthenogenesis (van Nouhuys and Ehrnsten 2004). No wasps emerged from the F031 treatment. Although this treatment was maintained for *M. cinxia* (Table S2), the data were later excluded from this study for simplicity and to allow direct comparison between species (Fig. 1). To investigate the failure of parasitoid development in the F031 treatment group, we dissected all deceased F031 caterpillars to check for the presence of dead *H. horticola*. When possible, we assessed the developmental stage at death using a Leica Z16 macroscope and a Flexacam C3 at the Finnish Museum of Natural History (LUOMUS).

#### Offspring generation (F1)

We divided all large butterfly egg clutches (>60 eggs) parasitised by *H. horticola* into two clutches of similar size. We randomly assigned each half of the clutches to the 26°C or 28°C temperature treatment. Small egg clutches (<60 eggs) parasitised by *H. horticola* were not split as young caterpillars kept in small groups have low survival (Kuussaari et al. 2004, Saastamoinen 2007, Duplouy and Hanski 2015). These small clutches were randomly assigned to the 26°C or 28°C temperature treatments. The butterflies in the F026 treatment only laid one small egg clutch, which was assigned to 28°C (Fig. 1). After breaking the diapause in the fall of 2021, postdiapause caterpillars from each temperature treatment were divided into smaller groups of 15–25 individuals that were randomly assigned to 26°C or 28°C temperature treatments until pupation and emergence of a parasitoid wasp or a butterfly host (Fig. 1). Adults (F1) from the final eight treatment groups (Fig. 1) were killed within 24 h after emergence by placing them at −20°C for 24 h. All specimens were transferred into individual Eppendorf tubes filled with 99% ethanol and labelled with their unique identification number.

### DNA extraction and sequencing

Each adult specimen was individually surface washed twice, by dipping it in two consecutive 10X-Phosphate-buffered saline (PBS) baths. The abdomen was cut off with a sterile blade and air-dried in a sterile hood environment for 2–4 h. DNA extractions were performed using a tissue kit on the Chemagic^TM^ 360 Nucleic Acid Extractor (PerkinElmer, USA) at the Molecular Ecology and Systematics (MES) Laboratory, at the University of Helsinki. We amplified the hypervariable V5–V6 bacterial region of the 16S ribosomal RNA (rrs) gene by Polymerase chain reaction (PCR) using primers 784F (5′-AGGATTAGATACCCTGGTA) and 1061R (5′-CRRCACGAGCTGACGAC) (Toft and Andersson 2010). Each specimen was amplified in duplicate. Each PCR reaction (18 µl total volume) contained 3 µl of DNA extract, 3.6 µl of 5x Q5 Reaction Buffer (New England Biolabs, USA), 3.6 µl of Q5 High GC Enhancer (New England Biolabs), 0.36 µl of dNTPs (2 mM), 0.1 µl of T4 gene 32 protein (10 mg/ml; New England Biolabs), 0.36 µl of bovine serum albumin, 0.36 µl of each primer (10 µM), 0.15 µl of Q5 High-Fidelity DNA Polymerase (2000 U/ml; New England Biolabs), and 6.95 µl of nuclease-free water. The PCR conditions were: 95°C for 5 min, followed by 40 cycles of 95°C for 1 min 40 s, 54.2°C for 1 min 45 s, 72°C for 1 min 40 s, and a final extension step of 72°C for 7 min. The PCR products of the same specimen were then pooled together (Duplouy et al. 2018, 2020), and sent for library preparation and sequencing at the Genomics NGS Sequencing unit, at the Finnish Institute for Molecular Medicine (FIMM), University of Helsinki. Sequencing was performed on a MiSeq v.3. Sequencing platform (Illumina, USA) using both reverse and forward primers. Raw sequencing data are available in NBCI under the accession code PRJNA1227207.

### Curation of amplicon sequencing data

To characterise the bacterial communities from *M. cinxia* and *H. horticola* samples in each temperature treatment, all 16S rRNA gene amplicons were processed using a home-developed pipeline based on USEARCH/VSEARCH (available at https://github.com/Symbiosis-JU/Bioinformatic-pipelines/blob/main/Example_analysis.md). Quality-filtered forward and reverse reads for both bins were assembled into contigs using PEAR (Zhang et al. 2014). Contigs were dereplicated (Rognes et al. 2016) and denoised (Edgar 2016). Chimeric sequences were removed after detection using USEARCH (Edgar 2010). The exact sequence variants (zOTU) of the 16S amplicons were clustered using the UPARSE-OTU algorithm implemented in USEARCH. The bacterial taxonomy was then assigned using the SINTAX algorithm and the SILVA database (version 138 SSU). Bacterial 16S rRNA gene amplicons were also screened for putative DNA extraction and PCR reagent contaminants, using negative controls (TE buffer blanks) as reference. After removing contamination, we normalised the data using Total Sum Scaling (TSS) and removed zOTU with a read count lower than 10 per sample.

### Statistical analyses

We used R version 4.4.0 (R Core Team 2018) for all statistical analyses. Data visualisation was done using the ggplot 2 3.4.4 package (Wickham 2011) in tidyverse 1.3.1 (Wickham et al. 2019).

To compare the bacterial communities associated with *M. cinxia* and *H. horticola*, we calculated Bray–Curtis, unweighted and weighted UniFrac distance matrices using the R package phyloseq 1.41.1 (McMurdie and Holmes 2013). In contrast with Bray–Curtis distance, both UniFrac distances account for the phylogenetic relatedness of zOTUs, but only the weighted UniFrac distance incorporates zOTU abundances (Lozupone and Knight 2005). To build the phylogenetic tree that was incorporated into the phyloseq object, we aligned the sequences and built a UPGMA tree (Sokal 1958) using MAFFT online service v7 (Katoh and Standley 2013, Katoh et al. 2019). Then, we tested for differences in bacterial composition using the permutational multivariate analysis of variance (PERMANOVA) (Anderson 2001) test using the function *adonis2* with 10 000 permutations. First, we tested the effects of insect species and generation as fixed explanatory variables, including the *M. cinxia* and *H. horticola* F0 and F1 samples. Because bacterial composition differed between species and generations, and because the sample size of F0 individuals was small (see Fig. 1), we subsequently focused our analyses on the effects of temperature treatments on the bacterial community of the F1 generation only, and separately for each insect species. Temperature was included in these models as a nested variable, with three levels: the temperature treatment experienced by F1 postdiapause caterpillars was nested within that of F1 prediapause caterpillars, which, in turn, was nested within the temperature treatment experienced by the F0 mother. Because life-history traits in *M. cinxia* are often found to differ among families (Verspagen et al. 2020), we added the IDs of the F0 mother butterflies or mother wasps as random effects in our models, passing them through the “strata” argument. For the *M. cinxia* models, sex was also added as a fixed effect. For *H. horticola*, because *Wolbachia* is temperature-sensitive (Hague et al. 2020, 2022), we conducted the analyses for *H. horticola* both using all samples and separately for individuals infected with *Wolbachia* and those not infected.

We compared the richness of bacterial species (zOTU richness) and the Shannon diversity index between species (*M. cinxia* versus *H. horticola*) and generations (F0 and F1) using generalised linear mixed models (GLMM) from the package glmmTMB 1.1.10 (Brooks et al. 2017). We first tested the effects of insect species and generation as fixed explanatory variables, including both the *M. cinxia* and *H. horticola* F0 and F1 samples. Then, we examined the effect of temperature on richness and Shannon index separately for the F1 specimens of each insect species, with the nested temperature treatments as fixed explanatory variables. We added the IDs of the F0 mother butterflies and mother wasps as random effects in the models. We used a Poisson distribution to model the richness of bacterial species (count data), and a Gaussian distribution to test variation in the Shannon index. For the *M. cinxia* models, sex was added as an additional fixed effect. For *H. horticola*, we performed the analyses considering all the samples and separately for individuals infected with *Wolbachia* and those that were not.

We selected the best model (PERMANOVA or GLMM) hierarchically, considering variable significance, and choosing the model with the lowest Akaike Information Criteria value, following the information-theoretic approach (Akaike 1998, Burnham and Anderson 2002). To identify bacterial taxa associated with each species and with specific temperature treatments, we conducted an indicator species analysis using the package indicspecies 1.8.0 (De Cáceres and Legendre 2009).

## Results

### Survival and developmental stages of parasitoids under exposure at 31°C

**Figure 2:**
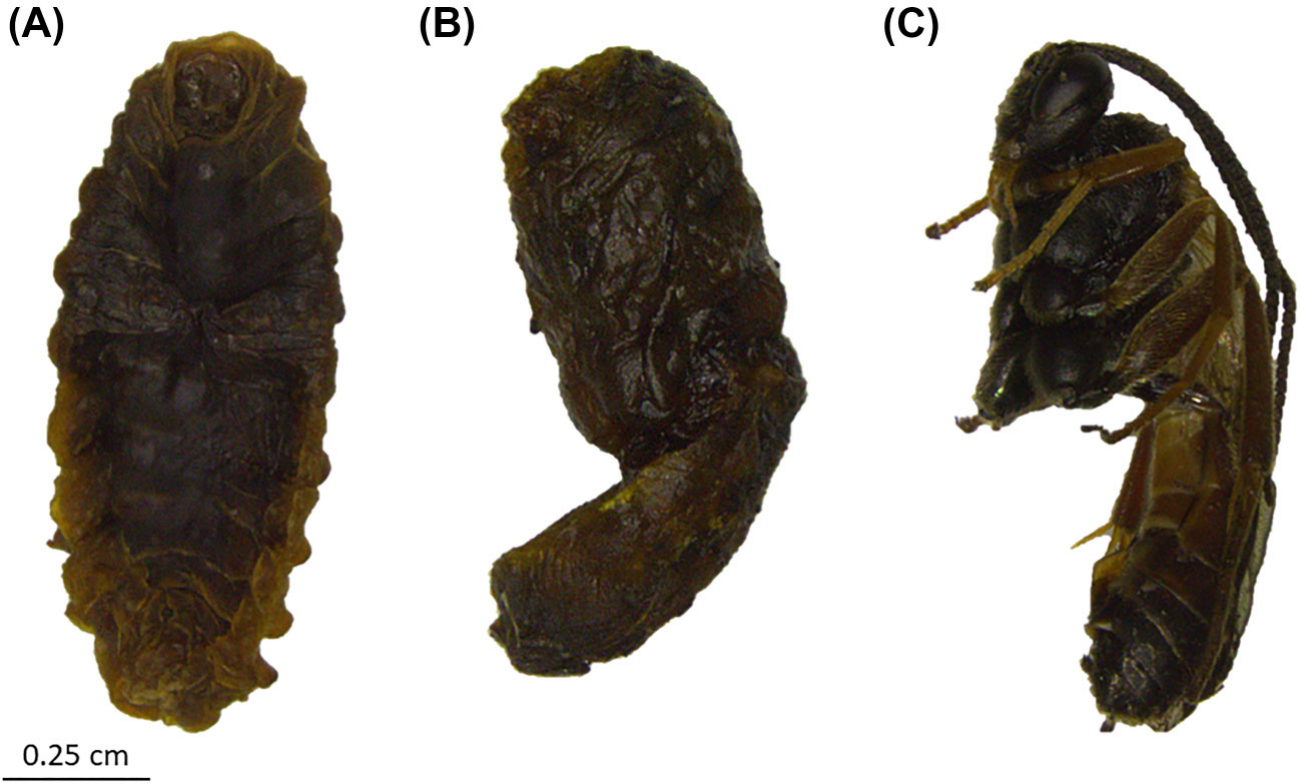
*Hyposoter horticola* exposed to the 31°C treatment died at three late stages of their development. (A) Parasitoid prepupa (specimen AF20-63-1). (B) Parasitoid pupa (specimen AF20-1025-5). (C) Pharate adult *H. horticola* (specimen AF20-6-12). The scale bar represents 0.25 cm for all images (A–C).

None of the F0 *H. horticola* exposed to the 31°C treatment survived. Dissection of the 30 caterpillars from that treatment that died revealed that 26 were clearly parasitised, while four did not contain a detectable parasitoid. Of the 26 parasitised hosts, 23 contained *H. horticola* and three were hyperparasitized by *Mesochorus* cf. *stigmaticus*. Among the *H. horticola*-parasitised individuals, one was found as a prepupa (Fig. 2A), seven as pupae (Fig. 2B) and 15 as pharate adults (Fig. 2C), indicating that the *H. horticola*-parasitised individuals died at a late development stage, after killing their hosts.

### Bacterial communities of *Melitaea cinxia* versus *Hyposoter horticola*

We obtained 1 479 zOTUs (2 879 567 reads) for the bacterial microbiota of *M. cinxia* and 4909 zOTUs (14 579 491 reads) for that of *H. horticola* before decontamination. After decontamination, 111 zOTUs (1 728 371 reads) remained for the bacterial community of *M. cinxia* and 129 zOTUs (13 780 536 reads) for that of *H. horticola*.

**Figure 3:**
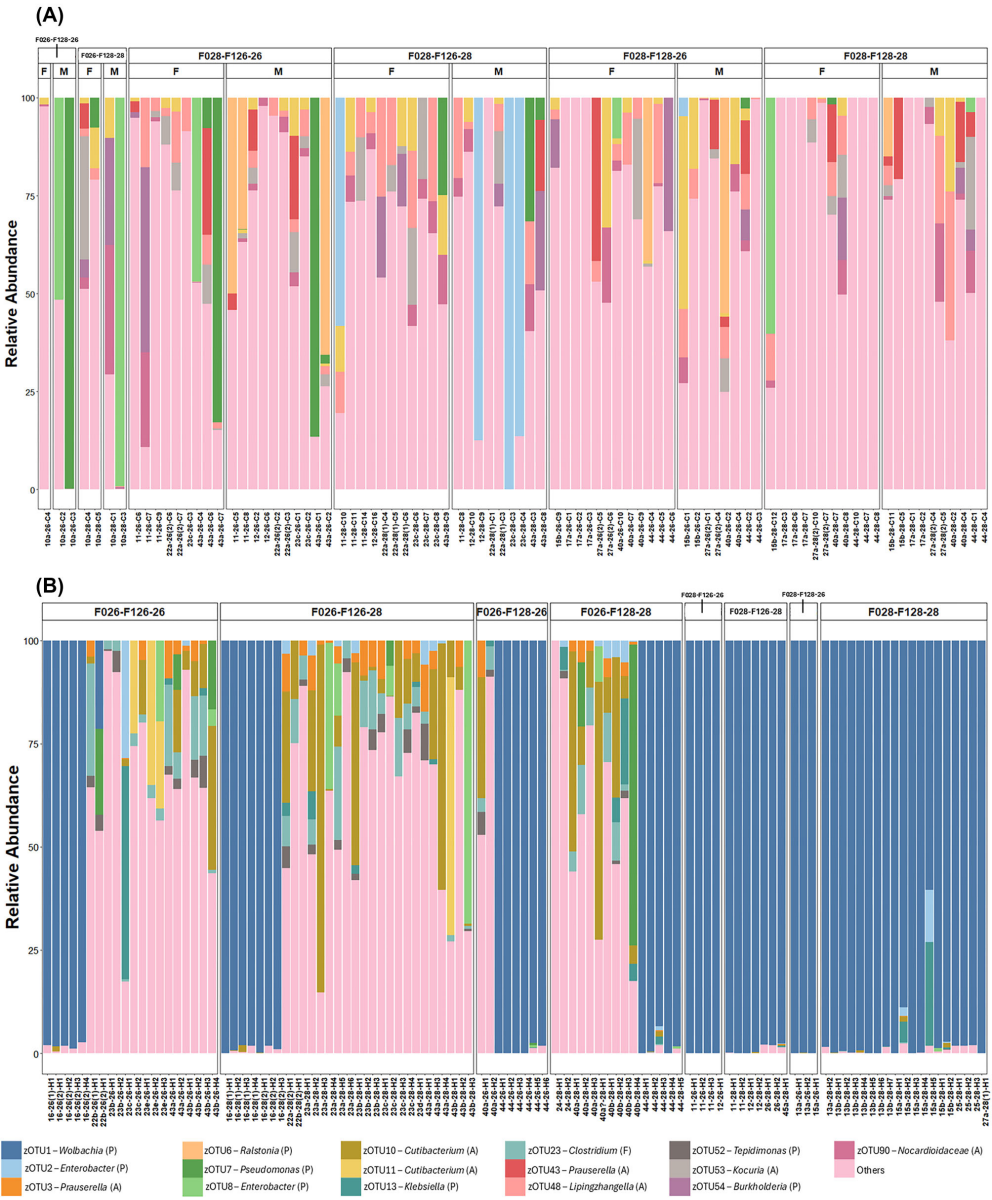
Stacked bar plot of the relative abundances of bacterial zOTUs in *M. cinxia* (A) and *H. horticola* (B). Each bar represents an insect specimen, with colours indicating the 10 most abundant zOTUs for each species. Letters preceding the zOTU names indicate bacterial phyla: A, Actinobacteriota; F, Firmicutes; and P, Proteobacteria. All other zOTUs are grouped as “Others”. Bars are grouped by temperature treatment, and for *M. cinxia* (A), they are further organised by sex (F, female and M, male).

The most abundant bacterial taxon detected in *M. cinxia* was *Enterobacter* (Proteobacteria) (zOTUs 2 and 8; Fig. 3A). However, this taxon was present in only 11% of all specimens, whereas the remaining individuals harboured a diverse mixture of bacterial taxa. Indicator species analysis identified 36 bacterial zOTUs significantly associated with *M. cinxia* and 50 zOTUs associated with *H. horticola* (Table S6). Among the strongest associations for *M. cinxia*, we observed, for instance, bacteria of the genera *Kocuria* (zOTU 43) and *Rubrobacter* (zOTUs 129 and 137) (indicspecies, *P* = .0001). In contrast, in *H. horticola*, the proteobacterium *Wolbachia* (zOTU 1) dominated the bacterial community of all specimens originating from F0 females reared at 28°C (F028), and approximately one-third of the specimens originating from F0 females reared at 26°C (Fig. 3B). Indicator species results for *H. horticola*, in addition to *Wolbachia*, included, for example, bacteria of the genera *Clostridium* (zOTU 23) and *Burkholderiales* (zOTU 73) (indicspecies, *P* = .0001).

**Figure 4:**
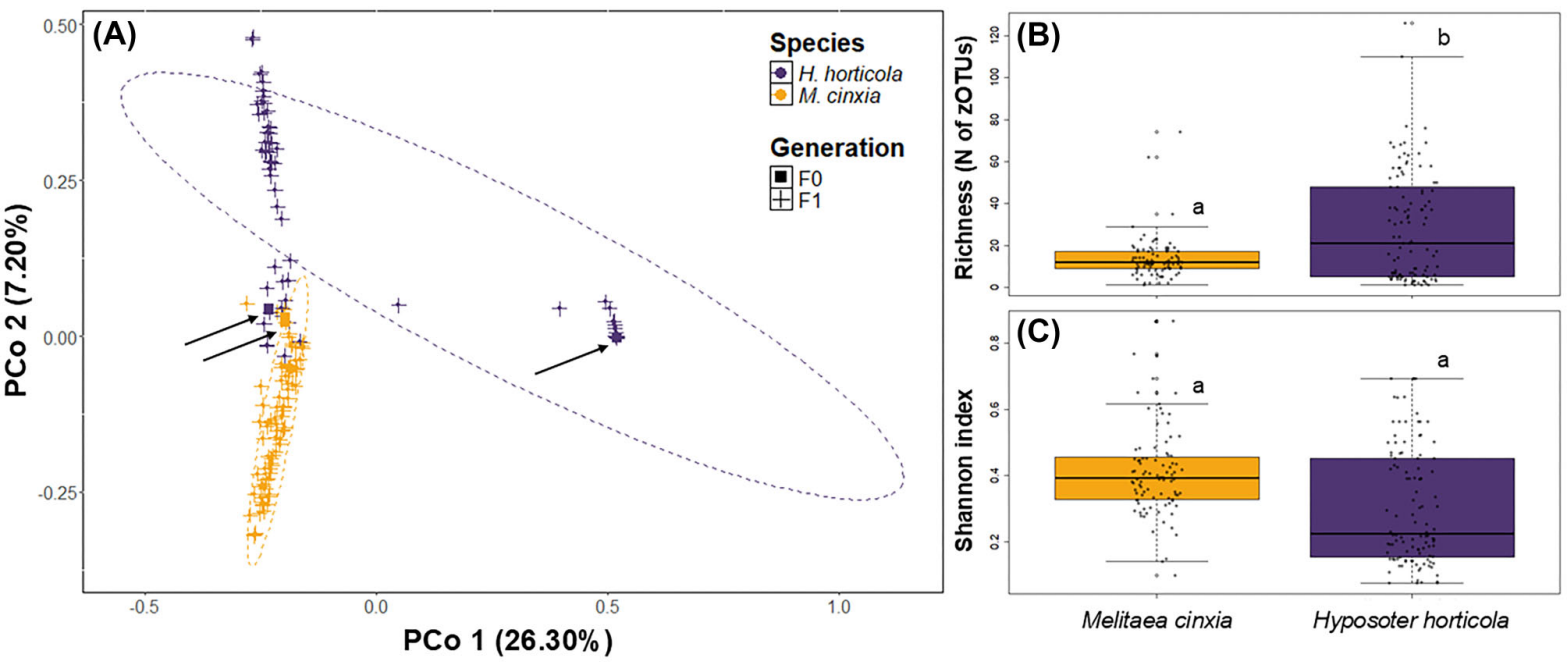
Microbiota composition, richness, and diversity for *M. cinxia* and *H. horticola*. (A) PCoA plot (Bray−Curtis distance) of parental (F0) (squares) and offspring (F1) (crosses) generations of *M. cinxia* (light colour) and *H. horticola* (dark colour) (*R*² = 0.124, *P* = .003). Ellipses indicate 95% confidence limits for each host species. Arrows highlight F0 specimens. (B) Bacterial species richness (N of zOTUs) and (C.) Shannon index for *M. cinxia* and *H. horticola*. Different letters above boxplots indicate significant differences among groups (*P* < .05).

The compositions of the bacterial community differed significantly between *M. cinxia* and *H. horticola* (PERMANOVA using Bray–Curtis dissimilarity: *R*² = 0.124, *P* = .003; Unweighted and weighted UniFrac distance metrics are presented in the Supplementary data) (Fig. 4A, Appendix S1, Table S4). However, both species shared 131 zOTUs, including zOTU 2 (*Enterobacter*, Proteobacteria), zOTU 7 (*Pseudomonas*, Proteobacteria), zOTU 8 (*Enterobacter*, Proteobacteria) and zOTU 11 (*Cutibacterium*, Actinobacteriota) (Fig. 3). The richness of bacterial species (number of zOTU) associated with the parasitoid was higher than that associated with the butterfly (GLMM: β = 0.324, *P* = .034) (Fig. 4B). However, the bacterial community of the two host species showed similar Shannon index values (GLMM: β = −0.041, *P* = .383) (Fig. 4C).

The bacterial communities of the parental generation (F0) differed significantly in composition from that of the generation of offspring (F1) for both *M. cinxia* and *H. horticola* (PERMANOVA Bray–Curtis: *R*² = 0.015, *P* = .025) (Fig. 4A, Table S4). However, there was no effect of generation on the richness of bacterial species or the Shannon index (Table S5).

### Bacterial communities of *M. cinxia* and *H. horticola* under temperature treatments

#### M. cinxia

**Figure 5:**
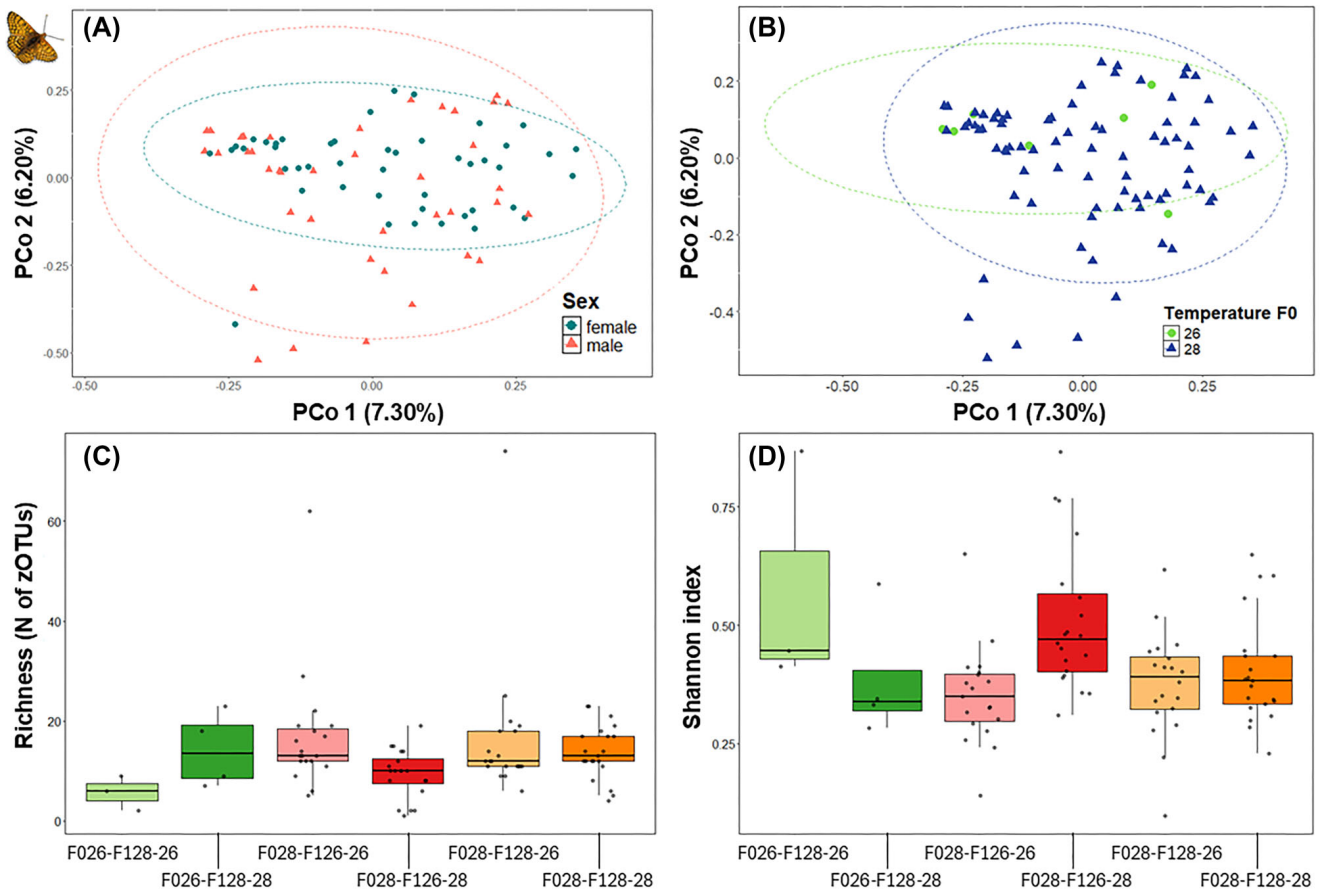
Microbiota composition, richness and Shannon index of adult *M. cinxia* F1. (A) PCoA plot based on Bray–Curtis distances, showing differences in the microbiota composition between sexes (female = circle, male = triangle) (*R*² = 0.016, *P* = .027). (B) PCoA plot using Bray–Curtis distances showing the difference in the microbiota composition according to the temperature experienced by the parental generation (F0) (26°C = circle; 28°C = triangle) (*R*² = 0.040, *P* = .102). (C) Richness (N of zOTU) and (D.) Shannon index across the temperature treatment groups (see Table S5 for statistical results).

The bacterial community composition in *M. cinxia* differed between sexes, with the microbiota of males showing greater dispersion/heterogeneity than that of females (PERMANOVA Bray–Curtis: *R*² = 0.016, *P* = .027) (Fig. 5A, Appendix S2A and C). However, no significant effects of the temperature experienced at the parental generation or during caterpillar pre- or postdiapause periods were observed on the bacterial composition of *M. cinxia* (Fig. 5B, Appendix S2B and D, Table S4).

The richness of bacterial species and the Shannon diversity index of the microbiota of *M. cinxia* did not differ between sexes (Appendix S3, Table S5), but were affected by temperature. The bacterial species richness of offspring (F1) was greater if the F1 samples were from mothers reared at 28°C compared to 26°C (GLMM: β = 1.088, *P* < .001). There were also significant three-way interactions between the temperatures of the parental, prediapause clutch, and postdiapause group, suggesting that different temperature combinations may influence the richness of bacterial species in adult butterflies in complex ways (Fig. 5C, Table S5). The Shannon index was lower for the offspring of mothers at high temperature (28°C) (GLMM: β = −0.223, *P* = .003), suggesting a reduced bacterial diversity when parents were exposed to higher temperatures. Finally, two significant three-way interactions between parental, prediapause, and postdiapause temperature treatments suggest that developmental stages may play a role in shaping bacterial diversity, as measured by the Shannon index (F028-F126-28, GLMM: β = 0.157, *P* < .001; F026-F128-28, GLMM: β = −0.189, *P* = .043) (Fig. 5D, Table S5).

Indicator species analysis for *M. cinxia* identified a small number of zOTUs associated with individual treatment groups (*N* = 9). However, these associations were uneven and limited to single treatments (Table S6). No indicator taxa were shared across temperature treatments, showing an absence of consistent treatment-specific bacterial associations.

#### H. horticola

##### Bacterial composition and diversity of *Wolbachia*-infected versus uninfected *H. horticola*

**Figure 6:**
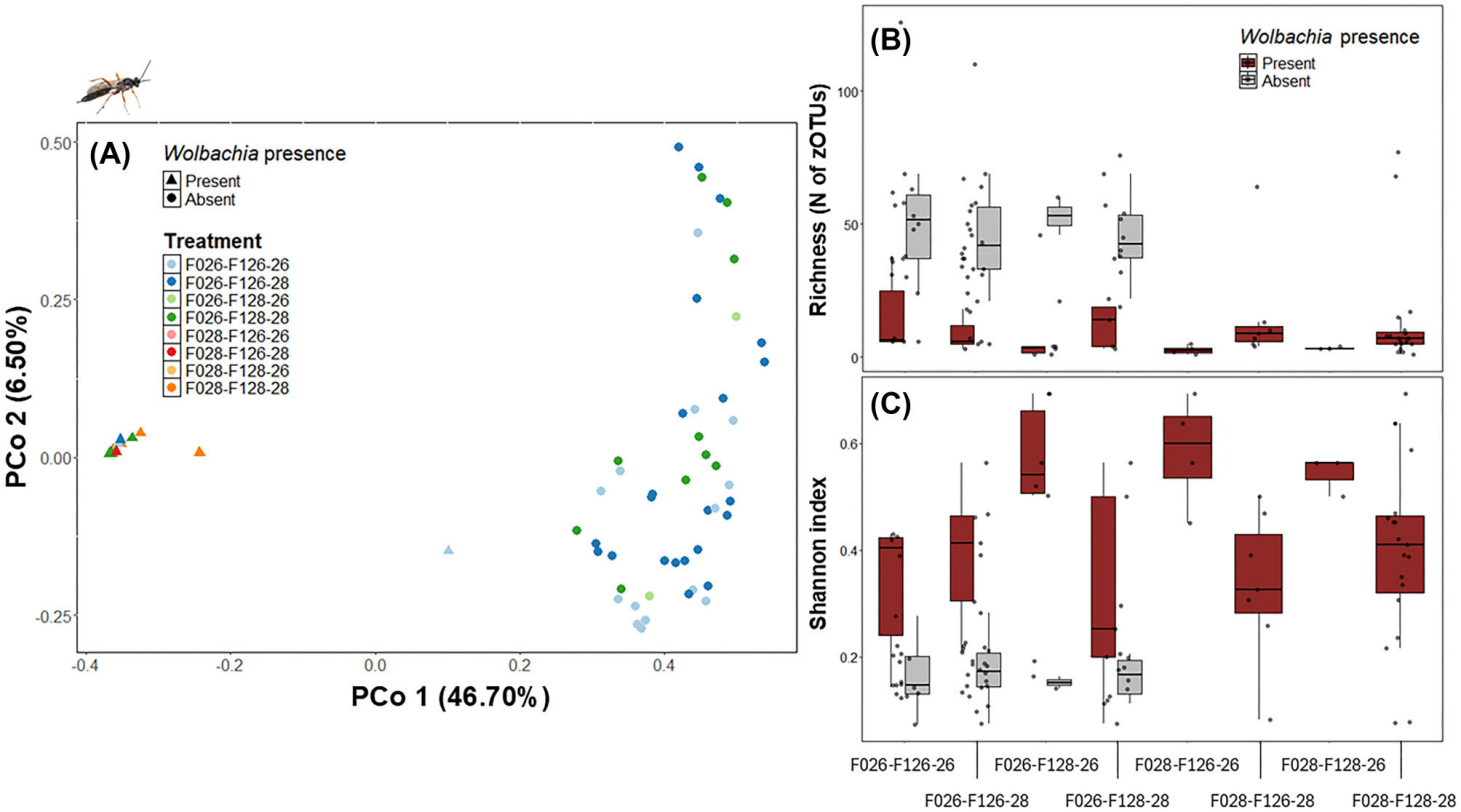
Microbiota composition, richness and Shannon index of adult *H. horticola* F1. (A) PCoA plot using the Bray–Curtis distance. Shapes represent individuals with or without *Wolbachia* (triangle = *Wolbachia* present; circle = *Wolbachia* absent) (*R*² = 0.455, *P* = .006), and colours indicate the eight temperature treatments (see Table S4). (B) Richness (N of zOTU) and (C.) Shannon index per temperature treatment for individuals with (dark colour) and without *Wolbachia* (light colour) (see Table S5 for statistical results).

The bacterial communities in the parasitoid wasp *H. horticola* were clearly divided into two types: those infected with the bacterium *Wolbachia* and those lacking the infection (Fig. 6A, Appendices S4 and S5, Table S4). None of the temperature treatments affected the composition of the bacterial communities associated with F1 *H. horticola* (Fig. 6A, Table S4).

The richness of bacterial species and the Shannon diversity index differed between *H. horticola* specimens, with individuals infected with *Wolbachia* exhibiting a lower richness of bacterial species and a higher Shannon index compared to those without *Wolbachia* (Fig. 6B and C, Appendix S6, Table S5). Furthermore, the samples infected with *Wolbachia* showed a wider dispersion in the Shannon index, indicating greater variability in the diversity of their associated bacterial communities, compared to the samples without *Wolbachia* (Fig. 6B and C, Table S5).

In *H. horticola*, the indicator species analysis revealed very limited support for treatment-specific bacterial associations, with only two bacteria, *Paracoccus* (zOTU 8812; indicspecies, *P* = .028) and *Wolbachia* (zOTU 17457; indicspecies, *P* = .027) identified as indicators of a single treatment group (F028-F128-26) (Table S6). No taxa were associated with the other treatments, suggesting that the diverse temperature treatments did not strongly select for consistent or distinct indicator communities.

##### Effects of temperature on bacterial diversity in *Wolbachia*-infected and uninfected *H. horticola*

*Wolbachia*-infected specimens exhibited lower bacterial species richness when their parents were reared at 28°C (GLMM: β = −2.179, *P* < .001). The effect of temperature in the parental generation on bacterial richness was further modulated by the temperatures experienced during different stages of the offspring development, with some treatments reducing richness and others increasing it (Table S5). In addition, the Shannon diversity index of *Wolbachia*-infected individuals was especially high when the parental generation had been reared at 28°C (GLMM: β = 0.253, *P* = .007). As with species richness, the effect of temperature at the parental generation on the Shannon index of the offspring bacterial community was influenced by temperature conditions across life stages, with some combinations reducing diversity and others enhancing it (Table S5).

In *Wolbachia*-free specimens, temperature played a role in shaping bacterial diversity. Individuals that experienced a prediapause clutch temperature of 26°C and postdiapause group temperature of 28°C had significantly reduced bacterial richness (GLMM: β = −0.137, *P* = .004) compared to other treatment groups. Conversely, exposure to 26°C during prediapause was associated with a higher Shannon index (GLMM: β = 0.163, *P* < .001).

## Discussion

By simultaneously exposing the Glanville fritillary butterfly (*M. cinxia*) and its specialist parasitoid (*H. horticola*) to different temperatures over two generations, we explored how species and temperature conditions at different developmental stages can shape the bacterial communities associated with a host and its parasitoid.

### Host–parasitoid thermal tolerance mismatch

*Melitaea cinxia* butterflies developed normally within the experimental range of 26°C–31°C, which are temperatures commonly experienced by this species under natural conditions in the Åland Islands (Verspagen et al. 2020). This butterfly species is also known to tolerate prolonged exposure to high temperatures, up to 34°C (Verspagen et al. 2020, 2023). Strikingly, at 31°C, the parasitoid died late in development, after killing its host, either during the final stages of pupation, or while attempting to emerge from the pupal case as an adult. This suggests that the late developmental stage might be the most sensitive stage for the wasp, and that 31°C may be within its upper thermal limit, potentially making this species more sensitive to climate change than its host butterfly. In the field, late-instar *M. cinxia* caterpillars, which are black, are known to bask in the sun, attaining temperatures far above 31°C (van Nouhuys and Lei 2004), which may be a host behavioural mechanism that could limit parasitoid development prior to parasitoid pupation and host death. Similar patterns between hosts and parasitoids have been observed in other systems. For example, survival of the parasitoid *Cotesia congregata* in its host *Manduca sexta* decreased with increasing temperatures, with complete mortality occurring at 30°C ± 10°C, whereas the unparasitized hosts remained unaffected (Moore et al. 2021, Malinski et al. 2023). Such a disparity in the thermal tolerance between host and parasitoids could serve as an enemy escape or enemy release strategy for host insects, allowing their persistence or range expansion in areas where their parasitoids might face decline as they reach their upper thermal tolerance limit (Malinski et al. 2024). As species respond uniquely to environmental stressors, thermal changes might disrupt ecological interactions and threaten parasitoid populations (Malinski et al. 2024). Characterising this discrepancy in the thermal limits between the parasitoid and its host might have some important consequences for the dynamics of such interactions.

### The bacterial communities of *M. cinxia* and *H. horticola* are host-specific

Although larvae of the parasitoid *H. horticola* feed and develop entirely within the *M. cinxia* caterpillar, the two species host distinct bacterial community composition and richness. This is consistent with other host–parasitoid studies, although these typically focus on the larval stage. For example, the bacterial microbiota of the larval parasitoids *Cotesia glomerata* and *Cotesia rubecula* differ in composition from that of their host caterpillars, *Pieris brassicae* and *Pieris rapae* (Gloder et al. 2024). However, such a difference between trophic levels was not found in a study by Hu et al. (2024) comparing the microbiota of *C. vestalis* larvae to that of their host, the moth *P. xylostella*, as both exhibited similar bacterial composition.

In our study, the bacterial communities associated with *M. cinxia* were dominated by an *Enterobacter* bacterium (Proteobacteria) and were also consistently associated with the indicator species of the bacterial genera *Kocuria* and *Rubrobacter*. Caterpillars of *M. cinxia* have previously been reported to harbour *Enterobacteriaceae*. This family comprises taxa commonly associated with animal guts and engaged in diverse host–microbe interactions, ranging from mutualistic to pathogenic (Minard et al. 2019). *Kocuria* are commonly described as environmental or plant-associated bacteria and have been reported from a range of insect hosts. These include the weevil *Rhynchophorus ferrugineus*, in which the bacteria show cellulose-degrading capacity (Basavand et al. 2024), cockroaches, where the bacteria exhibit antibacterial activity against *Staphylococcus aureus* (Alkhalifah 2021), and several moth species (Sevim and Sevim 2022). Similarly, *Rubrobacter* has been reported from the gut of the moth *Spodoptera frugiperda*, where it might play a putative nutritional role through the degradation of organic substrates (Rozadilla et al. 2020, Fu et al. 2023). In contrast, the bacterial microbiota of the parasitoid exhibited a higher number of zOTUs than *M. cinxia*, and the parasitoid’s bacterial community was characterised by the indicator species *Wolbachia, Clostridium*, and members of the *Burkholderiales*. In *H. horticola, Wolbachia* does not affect its host fecundity, longevity, nor dispersal (Duplouy et al. 2015). However, it has been shown to increase host susceptibility to hyperparasitism by *Mesochorus cf. stigmaticus* (van Nouhuys et al. 2016). Broader effects of *Wolbachia* on parasitoid population dynamics (Duplouy et al. 2021), as well as its sensitivity to temperature, remain unclear. *Clostridium* has been reported across a wide range of insect taxa (Douillard et al. 2025), and although some *Clostridium* species are pathogenic to vertebrates, insects could act as vectors in the environment or benefit from their presence through improved digestion or protection against pathogens (Douillard et al. 2025). Finally, bacterial members of *Burkholderiales* are environmentally acquired symbionts known to confer nutritional benefits, resistance to insecticides, or protection against pathogens in stinkbugs, beetles, and ants (Kaltenpoth and Flórez 2020, Stillson et al. 2022). Together, these results suggest that the indicator taxa identified in *M. cinxia* and *H. horticola* may play roles in host nutrition and host protection, although these functions remain to be further tested.

We observed significant differences in community composition between the bacterial microbiota of F0 and F1 generations in both the butterfly and parasitoid, independent of temperature effects. This pattern was indeed expected, since wild F0 and lab-reared F1 insect populations experienced different diets and rearing environments, which are known to strongly influence insect-associated bacterial communities (Tinker and Ottesen 2021). However, interpretation of F0-associated microbiota patterns is necessarily limited because the F0 sample sizes were small due to substantial mortality across developmental stages and limited successful mating or parasitism events, resulting in insufficient replication for robust statistical inference. Consequently, F0 data were used solely to assess broad generational differences. We also noted that in *M. cinxia*, the bacterial community differed between the sexes and showed greater dispersion (i.e. heterogeneity) in males than in females. However, bacterial species richness and diversity remained similar in both sexes. Therefore, while males and females harbour similar numbers of bacterial taxa, the composition of the community differs, with higher variability in the bacterial communities between male host individuals. These results contrast with previous research on *M. cinxia*, which found no differences between sexes (Minard et al. 2019). However, the study by Minard et al. (2019) analysed the bacterial microbiota of caterpillars, whereas our study examined the microbiota of adult butterflies. Investigating whether the composition of the bacterial microbiota also differs between *M. cinxia* and *H. horticola* at the larval stage, and when such differences could arise during male and female development, are important next steps.

In the parasitoid, *Wolbachia* dominated the bacterial microbiota of all F1 specimens originating from F0 females reared at 28°C (F028), and approximately one-third of those originating from F0 females reared at 26°C (F026). The higher prevalence observed in F028-derived offspring reflects the maternal infection status, as by chance all three F028 mothers carried *Wolbachia*, whereas only one of the two F026 mothers was infected. This pattern is consistent with maternal transmission of *Wolbachia* and aligns with previous findings showing that *Wolbachia* occurs in approximately half of *H. horticola* individuals in natural populations from the Åland Islands, Finland (Duplouy et al. 2015, 2021). However, as a limitation of this study, the small number of F0 females prevented us from obtaining uninfected mothers at 28°C. Finally, because all F1 *H. horticola* individuals in our study were male, we were unable to assess sex-specific effects on the composition, richness, or diversity of their bacterial microbiota, which represents an additional limitation of this study.

In *H. horticola*, the composition, richness, and diversity of the bacterial microbiota differed depending on the presence or absence of the *Wolbachia* bacterium (zOTU 1). We observed that the presence of *Wolbachia* in *H. horticola* is associated with low bacterial richness but high Shannon diversity. As a highly abundant symbiont, *Wolbachia* may compete with other bacteria for resources, potentially reducing the number of rare taxa and leading to a more even distribution in the remaining bacterial taxa (Li et al. 2022). However, because we lack absolute bacterial density estimates, inferring resource competition is not possible. Alternatively, these patterns may reflect sequencing artefacts (Wilches et al. 2021), limiting our power to interpret this data.

### Temperatures at the parental generation affect bacterial richness in the offspring for both the host and parasitoid

There was little to no effect of temperature treatments on the overall composition of the bacterial microbiota for *M. cinxia* or *H. horticola*. However, bacterial richness and Shannon index were significantly affected by the temperature experienced by the parental generation (F0), as well as by temperature treatments in the different larval stages (pre- vs postdiapause larval stage) of the offspring generation (F1).

In both *M. cinxia* and *H. horticola*, the thermal conditions experienced by the maternal generation (F0) influenced the microbial species richness and the Shannon index. In *M. cinxia*, the richness was higher and the Shannon index was lower when the mothers were reared at 28°C. In contrast, the opposite effect was observed in *H. horticola*: the richness was lower and the Shannon index was higher when the mothers were reared at 28°C compared to 26°C. This suggests that the effects of environmental conditions manifest themselves transgenerationally. If offspring inherit a portion of their bacterial microbiota from their mother (e.g. through egg coating, transovarial transmission, or gut symbionts passed at oviposition), thermal stresses on mothers could shape the initial maternally transmitted bacterial community, which then persists or influences the later development of the offspring microbiota (Guilhot et al. 2023). Alternatively, thermal conditions may affect how the bacterial microbiota of the offspring assembles during early development and after the maternal transmission of the microbes. If the assembly of the bacterial community is more plastic during the early stages of development (when the offspring microbiota is first formed), the temperature experienced by mothers might shape the bacterial community in a way that persists throughout development (Guilhot et al. 2023). To our knowledge, only one study has investigated the transgenerational effects of the insect bacterial microbiota under different temperature treatments. This study, conducted using the cockroach *Diploptera punctata*, also found that temperatures experienced by the parental generation had a greater effect on the bacterial community associated with the offspring than the temperature experienced by the offspring themselves (Bridson 2020). Future studies could investigate the mechanisms by which parental temperature influences the adult bacterial microbiota of the offspring in the *M. cinxia–H. horticola* association. Although there were interactions between temperatures at different developmental stages, no single stage appeared to be more sensitive to temperature than the others. This suggests that temperature might have some effect during development, but these effects are not general and would be difficult to interpret without a more focused study.

The lower richness and higher Shannon diversity observed in *H. horticola* when mothers were reared at 28°C (compared to 26°C) can be explained by the presence of *Wolbachia*. This is supported by the stronger effects of temperature treatments on both bacterial richness and Shannon index in *Wolbachia*-infected individuals compared to uninfected ones. These patterns may occur because *Wolbachia* is more sensitive to temperature fluctuations (Arnold et al. 2019, Hague et al. 2020, Lau et al. 2020) than other bacterial taxa within the parasitoid. This finding aligns with previous research demonstrating that *Wolbachia* density is temperature dependent (Hague et al. 2020, 2022). Temperature-driven changes in *Wolbachia* abundance likely influence the overall richness of the parasitoid’s bacterial microbiota and the abundance of the other bacterial species (Li et al. 2022). Again, because our dataset does not include absolute abundance measurements, this interpretation should be taken with caution. Nonetheless, altogether our results underscore that insect hosts and their parasitoids have distinct bacterial communities, which respond differently to thermal conditions, especially those experienced at the parental generation.

## Supplementary Material

fiag046_Supplemental_Files

## Data Availability

Raw sequences are available in NCBI through the accession code PRJNA1227207.
