# Supplementary material for: Temperature at parental generation affects bacterial communities associated with offspring for both host and parasitoid
Source: FEMS Microbiol Ecol. 2026 May 1;102(6):fiag046. doi: 10.1093/femsec/fiag046 (PMC13192552; doi:10.1093/femsec/fiag046)
Supplement: fiag046_Supplemental_Files [file fiag046_supplemental_files.zip › Temperature_microbiota_SupplementaryFigures.docx]

# Supplementary figures

## Appendix S1


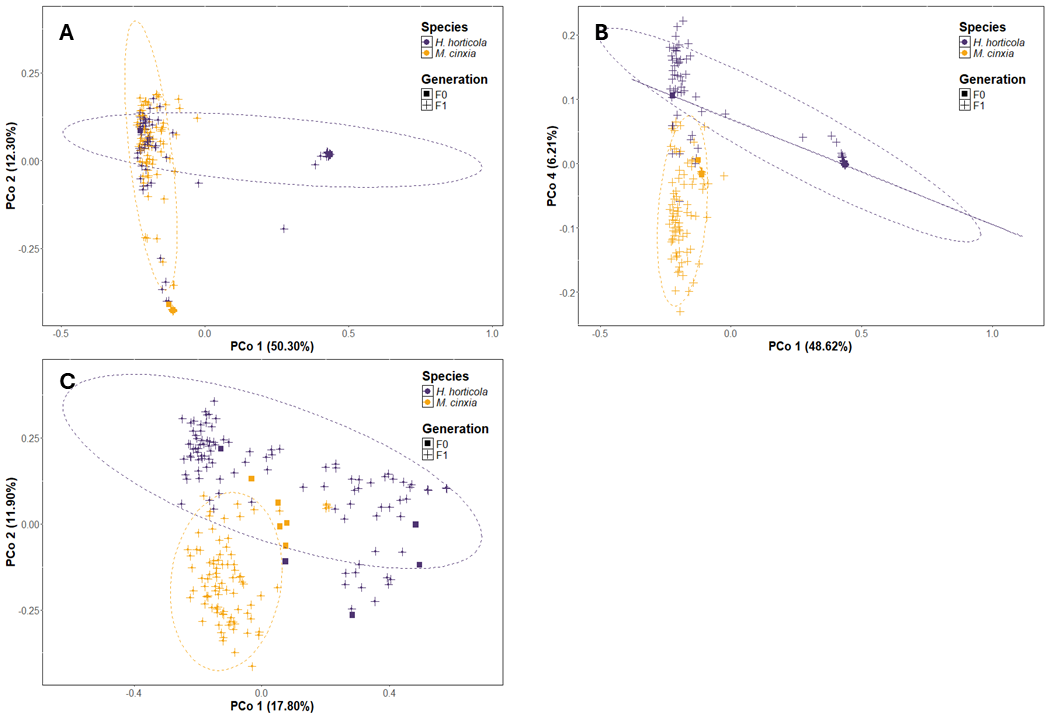


Figure S1. PCoA plots of parental (F0) (squares) and offspring (F1) (crosses) generations of *M. cinxia* (yellow) and *H. horticola* (purple). A. Weighted UniFrac distance PCoA plot (axes 1 and 2). B. Weighted UniFrac distance PCoA plot (axes 1 and 4). C. Unweighted UniFrac distance PCoA plot.

## Appendix S2


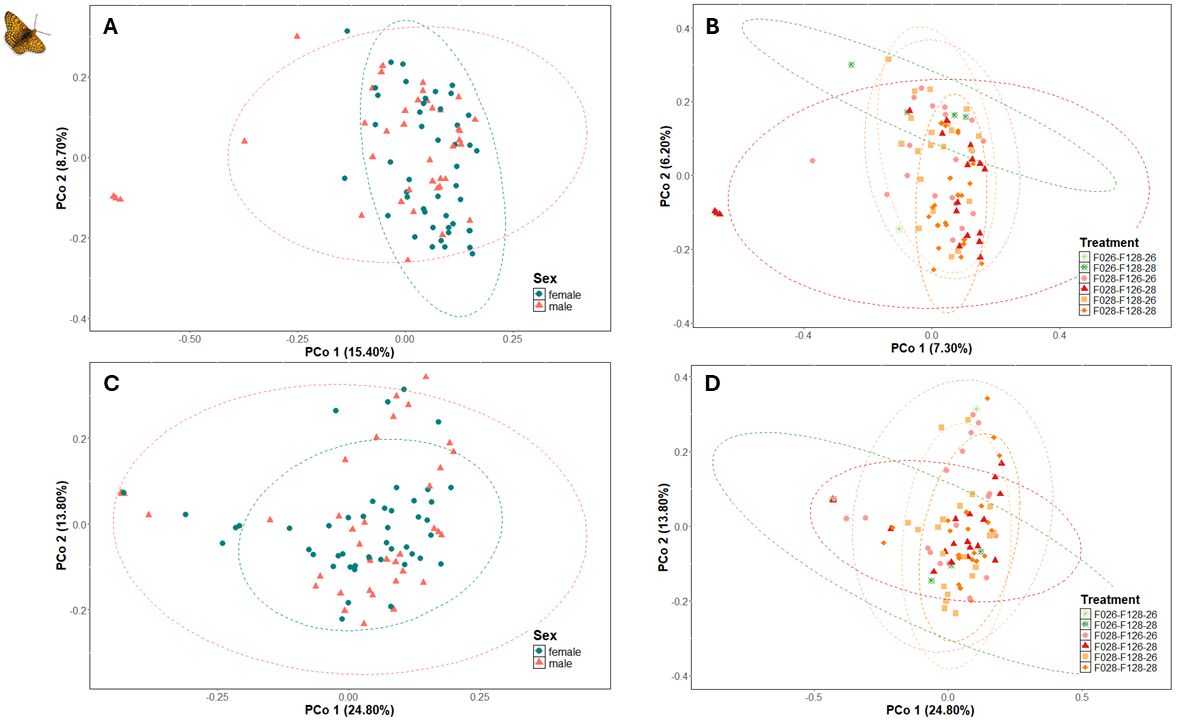


Figure S2. PCoA plots of *Melitaea cinxia* offspring generation (F1). A. PCoA plot using Unweighted UniFrac distance colored by sex (female = blue; male = orange) and (B.) by temperature treatment. C. PCoA plot using Weighted UniFrac distance colored by sex (female = blue; male = orange) and (D.) by temperature treatment.

## Appendix S3


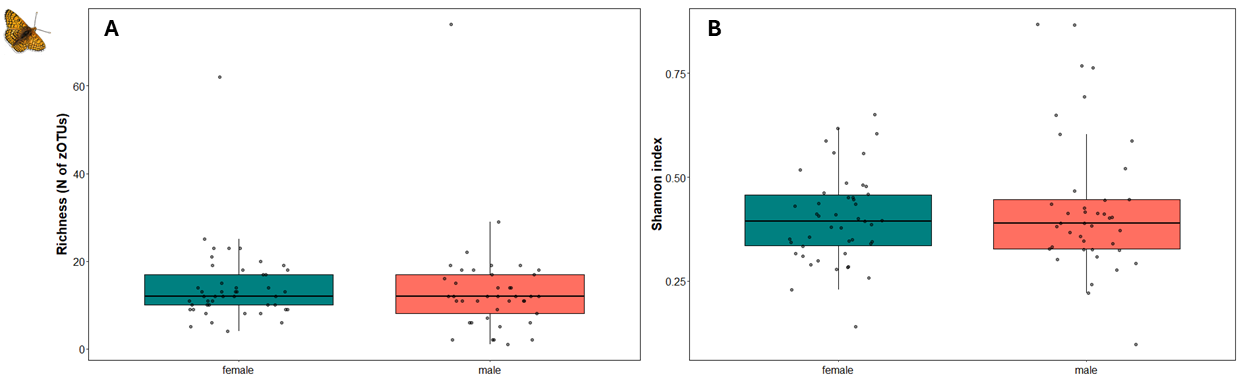


Figure S3. Richness and Shannon index for *Melitaea cinxia*. A. Richness (N of zOTUs) and (B.) Shannon index per sex for the offspring (F1) of *M. cinxia.*

## ***Appendix S4***


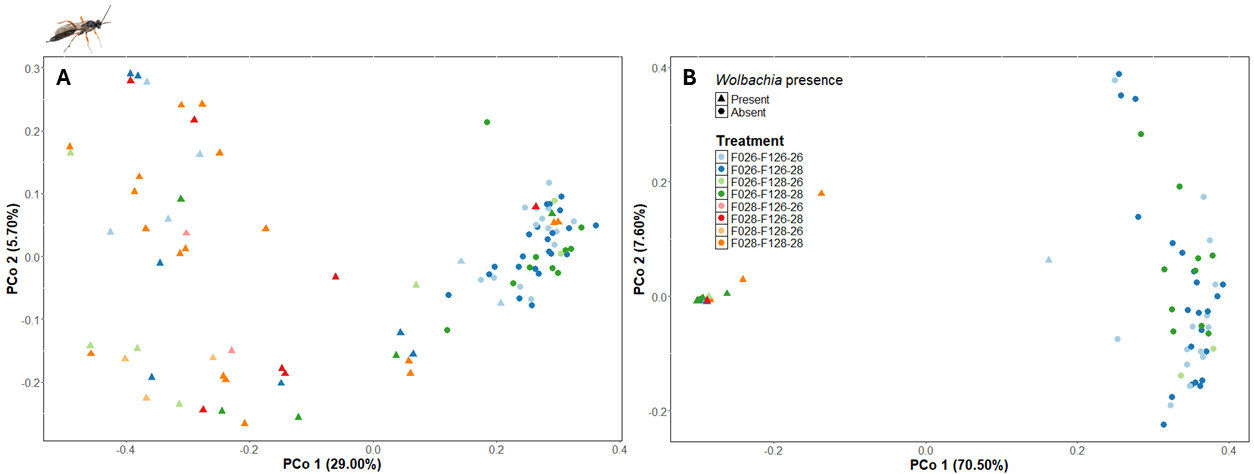


Figure S4. Microbiota composition of F1 adult *Hyposoter horticola* specimens. A. PCoA plot using unweighted UniFrac distance. B. PCoA plot using weighted UniFrac distance. Shapes represent individuals with or without *Wolbachia* (triangle = *Wolbachia* present; circle = *Wolbachia* absent), and colors indicate the eight temperature treatments.

## ***Appendix S5***


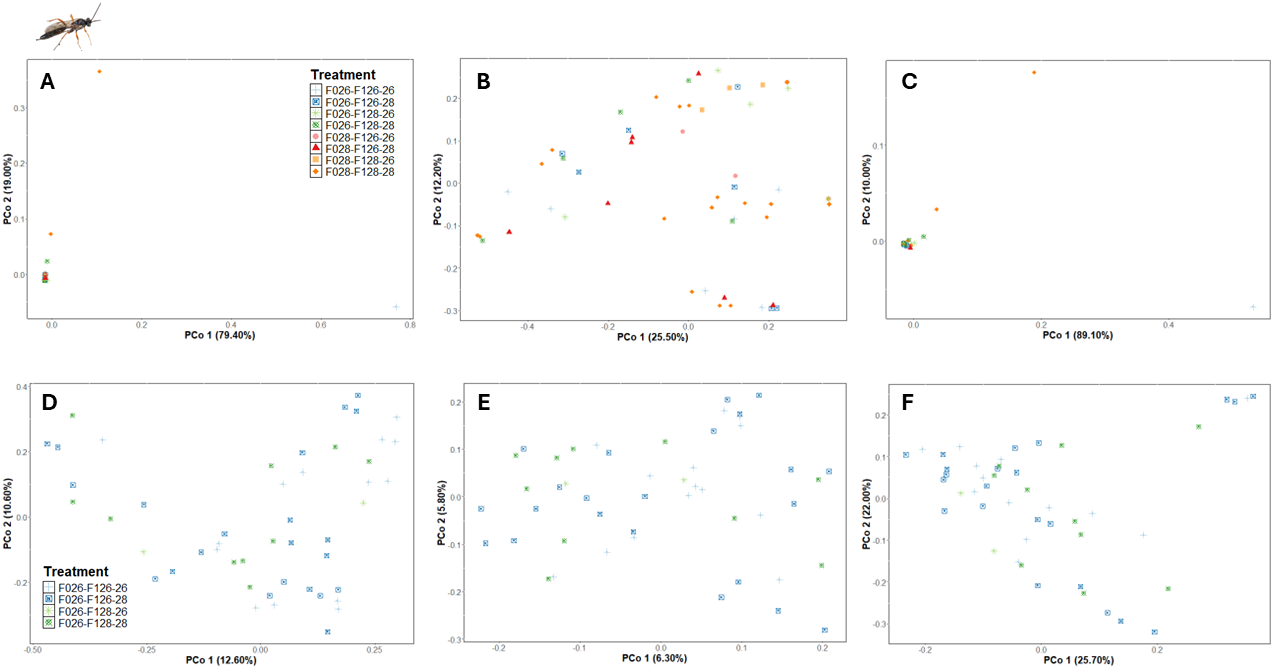


Figure S5. Microbiota composition of F1 adult *Hyposoter horticola* specimens colored by temperature treatment for individuals with and without *Wolbachia*. A-C: *Wolbachia* present. D-F: *Wolbachia* absent. A, D. Bray-Curtis distance. B, E. Unweighted UniFrac distance. C, F. Weighted UniFrac distance.

## ***Appendix S6***


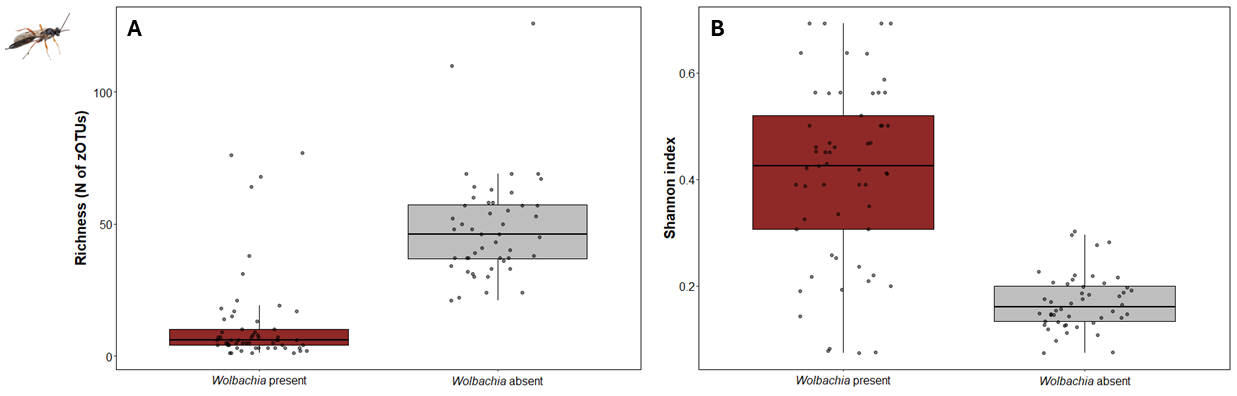


Figure S6. Richness and Shannon index for *Hyposoter horticola*. A. Richness (N of zOTUs) and (B.) Shannon index for offspring (F1) individuals with and without *Wolbachia* of *H. horticola.*
